# Evaluating Modern Therapeutic Interventions for Migraine Management: A Systematic Review

**DOI:** 10.7759/cureus.67397

**Published:** 2024-08-21

**Authors:** Lovett S Achiatar, Iqra Nasir, Zainab Zia, Hind Jameel, Yogesh Raut, Hamza Sher, Abdullah Shehryar, Benazir Shafqat, Khadija A Palekar, Lyba Nisar, Abdur Rehman, Moosa Khan

**Affiliations:** 1 Acute Medicine, Medway Maritime Hospital, Kent, GBR; 2 Internal Medicine, Islamic International Medical College, Rawalpindi, PAK; 3 Emergency Medicine, Kurdistan Regional Government, Erbil, IRQ; 4 Internal Medicine, NKP Salve Institute of Medical Sciences, Nagpur, IND; 5 Internal Medicine, Allama Iqbal Medical College, Lahore, PAK; 6 Emergency Medicine, Saudi German Hospital, Khamis Mushait, SAU; 7 Internal Medicine, Fortis Hospital, Mumbai, IND; 8 Internal Medicine, Quaid-e-Azam Medical College, Bahawalpur, PAK; 9 Surgery, Mayo Hospital, Lahore, PAK; 10 General Surgery, Nishtar Medical University, Multan, PAK

**Keywords:** acute migraine management, myofascial release, cgrp antagonists, non-pharmacological treatments, pharmacological treatments, migraine disorders

## Abstract

This systematic review evaluates the efficacy and safety of contemporary migraine treatments, synthesizing evidence from recent randomized controlled trials (RCTs). The focus is on both pharmacological interventions, such as calcitonin gene-related peptide (CGRP) monoclonal antibodies and non-specific oral migraine preventives, and non-pharmacological approaches like myofascial release. Through a detailed examination of the studies, this review identifies superior strategies for acute and preventive migraine management, assessing their impact on patient-reported outcomes and determining the prevalence of associated adverse events. Findings suggest that while CGRP monoclonal antibodies show promise as first-line treatments due to their efficacy and safety, myofascial release offers considerable benefits for pain and disability in tension-type and cervicogenic headaches. Challenges such as the variability in individual response and potential side effects emphasize the need for personalized treatment plans. This review underscores the importance of integrating new therapeutic discoveries into clinical practice to enhance the quality of care for migraine sufferers.

## Introduction and background

Migraine remains one of the most common and debilitating neurological disorders worldwide, affecting millions of individuals and imposing significant burdens on both healthcare systems and society. The complexity of migraine pathophysiology has led to the development of a diverse range of therapeutic strategies, each aiming to mitigate the frequency and severity of migraine episodes [1]. Recent advances in migraine management have seen the introduction of novel pharmacological agents such as calcitonin gene-related peptide (CGRP) antagonists [2] and innovative non-pharmacological treatments like myofascial release techniques [3]. However, the rapid evolution of therapeutic options necessitates a rigorous assessment of their efficacy and safety to guide clinical practice. Furthermore, the variability in individual responses to these treatments highlights the need for personalized treatment plans [4]. This systematic review aims to synthesize current evidence from recent randomized controlled trials (RCTs) to evaluate the effectiveness and safety of modern therapeutic interventions for migraine, providing a critical comparison across different treatment modalities.

The primary objective of this systematic review is to compare and contrast the efficacy and safety of various contemporary migraine treatments, including pharmacological interventions such as CGRP monoclonal antibodies and non-specific oral migraine preventives, as well as non-pharmacological approaches like myofascial release. This review aims to synthesize findings from randomized controlled trials to identify superior treatment strategies for both acute and preventive migraine management. The focus will be on assessing the impact of these treatments on patient-reported outcomes and the prevalence and nature of any associated adverse events. By compiling and analyzing existing research, this comprehensive evaluation will inform best practices and support healthcare providers in making evidence-based decisions tailored to individual patient needs, ultimately enhancing the quality of care for migraine sufferers.

## Review

Materials and methods

Search Strategy

Our systematic review was meticulously structured to align with the Preferred Reporting Items for Systematic Reviews and Meta-Analyses (PRISMA) guidelines, aimed at evaluating the effectiveness and safety of migraine therapies. We conducted an extensive search through major databases, including PubMed, MEDLINE, Embase, the Cochrane Library, and Web of Science, covering literature from the databases' inception until March 2024. This extensive timeframe allowed for a comprehensive capture of relevant studies.

We employed a strategic combination of keywords and Medical Subject Headings (MeSH) terms such as "migraine disorders," "pharmacological treatments," "non-pharmacological treatments," "CGRP antagonists," "myofascial release," and "randomized controlled trials." Boolean operators were utilized to integrate these terms efficiently, crafting search strings like "migraine disorders AND CGRP antagonists AND clinical efficacy" and "acute migraine treatment OR non-pharmacological interventions AND randomized trials." Our strategy also included a review of reference lists from selected articles and searches of clinical trial registries and conference proceedings to include unpublished or ongoing studies. An expert in neurological disorders refined our search strategy, ensuring the inclusion of peer-reviewed studies published in English focusing on adult migraine management. This rigorous method guarantees a thorough evaluation of modern migraine interventions, maintaining academic rigor and practical relevance in clinical settings.

Eligibility Criteria

Our systematic review established stringent eligibility criteria to ensure the inclusion of studies with high methodological quality and relevance to adult migraine management. We focused exclusively on peer-reviewed research articles, including clinical trials, RCTs, and meta-analyses that adhere to the International Classification of Headache Disorders (ICHD) criteria. Eligible studies are needed to evaluate pharmacological treatments like CGRP antagonists, triptans, and NSAIDs, or non-pharmacological approaches such as myofascial release and behavioral therapy. Each study was required to include comparative analyses against placebos, no treatment, or other active treatments, and report outcomes like reductions in migraine frequency, intensity, disability, quality of life, and safety profiles. Only studies published in English up to March 2024 were considered to ensure contemporary relevance.

Exclusion criteria were carefully delineated to sharpen the review’s focus and maintain its clinical applicability. We excluded studies that did not directly investigate therapeutic interventions for migraine management, those involving pediatric or non-adult populations, and research based on animal models. In addition, gray literature such as conference abstracts, unpublished works, and non-English language studies were omitted to ensure a focus on robust and directly applicable human patient outcomes. This selective approach was critical in upholding the scientific rigor and ensuring the depth and integrity of our systematic review, aiming to provide a comprehensive and clinically valuable synthesis of the current landscape in migraine treatment.

Data Extraction

Our data extraction protocol was meticulously crafted to ensure the reliability and validity of the information gathered for our systematic review of contemporary therapeutic interventions for migraine. The process began with a preliminary screening where two independent reviewers evaluated articles based on titles and abstracts, classifying them as "relevant," "not relevant," or "probably relevant." This crucial step allowed us to pinpoint the studies most pertinent to our review's focus.

For articles advancing past initial screening, a detailed full-text review was conducted. We employed a standardized data extraction form in Microsoft Excel (Microsoft Corporation, USA) to ensure uniformity and rigor during this phase. Reviewers filled out this form independently for each article, strictly adhering to our predefined inclusion and exclusion criteria. Discrepancies between reviewers were resolved by a third, mediating reviewer to maintain the accuracy and consistency of our data collection. This form captured essential details such as the lead author’s name, publication year, study design, population size, intervention specifics, key findings, and any limitations, enabling a comprehensive analysis and facilitating a nuanced discussion of the results within the broader context of migraine management literature and clinical practice.

Data Analysis and Synthesis

Given the diversity in therapeutic interventions and the varying clinical outcomes across studies, a meta-analysis was considered unsuitable due to significant heterogeneity. Instead, our review concentrated on a qualitative assessment and synthesis of findings, allowing an in-depth examination of each therapeutic intervention for migraine management, assessing their efficacy and safety across different patient demographics and migraine conditions.

We organized the key findings from each study to discern overarching themes and notable variations in treatment efficacy, safety profiles, and patient-reported outcomes. This thematic analysis revealed consistent, effective practices and discrepancies in treatment responses, enhancing our understanding of migraine management strategies. Our narrative synthesis compiled these insights to provide a holistic view of the current migraine therapy research landscape. We explored the implications of these findings, identified research gaps, and suggested future research directions. This approach not only highlighted the data relationships and differences but also critically evaluated the evidence's robustness and validity, offering a nuanced perspective on the efficacy and safety of existing migraine treatments and supporting the development of refined targeted therapeutic strategies.

Results

Study Selection Process

**Figure 1:**
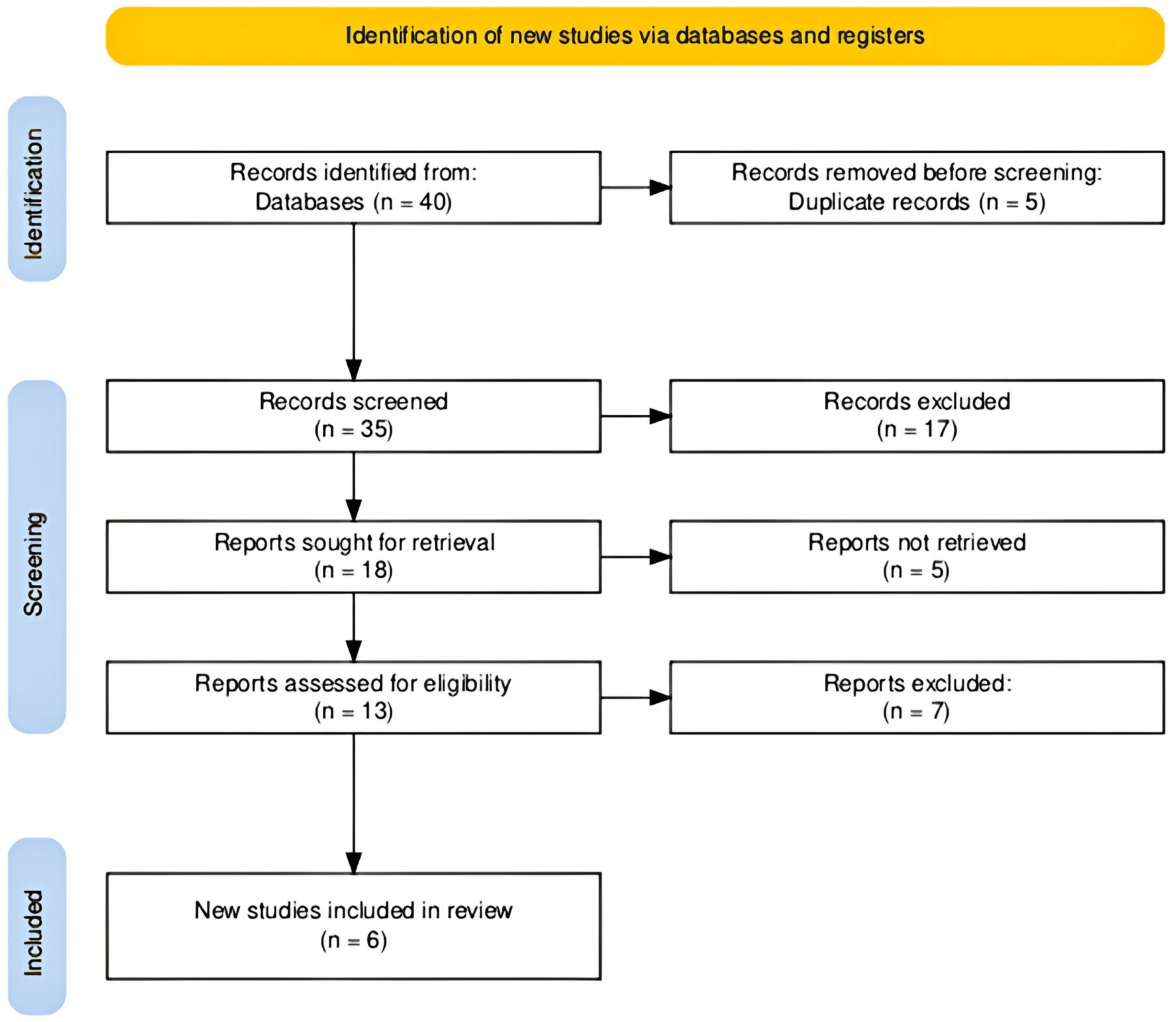
The PRISMA flowchart illustrating the study selection process. PRISMA: Preferred Reporting Items for Systematic Reviews and Meta-Analyses

The search across multiple databases yielded a total of 40 records for our systematic review of therapeutic interventions for migraine. After the removal of five duplicate records, 35 records were screened. This initial screening led to 18 reports being selected for more detailed evaluation. Of these, 13 reports were fully assessed for eligibility based on our predefined criteria. Following this thorough assessment, six studies were ultimately deemed suitable and included in our review. The entire study selection process is efficiently visualized in the provided PRISMA flowchart in Figure 1, ensuring clarity and transparency of our methodological approach.

Characteristics of Selected Studies

Our systematic review incorporates seven key studies, meticulously documenting the efficacy and safety of various migraine management therapies. Robblee et al. [2] provided a comparative analysis of CGRP monoclonal antibodies against traditional treatments, advocating their use as first-line preventives. Lu et al. [3], employing meta-analytic methods on diverse databases up to 2023, showcased the effectiveness of myofascial release in alleviating pain and disability from tension-type and cervicogenic headaches, with mixed outcomes for migraine. Deng et al. [5] conducted a 2023 network meta-analysis that evaluated novel treatments like lasmiditan, rimegepant, and ubrogepant, demonstrating the latter two's favorable profile for acute migraine management. Fernández-Bravo-Rodrigo et al. [6] assessed the real-world effectiveness of erenumab, noting significant reductions in migraine frequency and severity. Suresh et al. [7] analyzed the acute treatment efficacy of zavegepant in 2023, revealing its superiority over placebo albeit with an increase in adverse events. Lastly, Kirkland et al. [8], through a network approach, highlighted the benefits and risks of parenteral agents in emergency settings. Each study, robust in findings, acknowledged inherent limitations such as potential biases and heterogeneity across study designs. A summary of these pivotal studies is detailed in Table 1.

**Table 1:** Summary of all the principle studies included in the review. MFR: myofascial release, TTH: tension-type headache, CGH: cervicogenic headache, RCT: randomized controlled trial, CGRP: calcitonin gene-related peptide, mAbs: monoclonal antibodies, NOEPs: nonspecific oral migraine preventives, MMD: monthly migraine days, PROSPERO: International Prospective Register of Systematic Reviews

Authors	Objective	Design	Methods	Results and conclusions
Robblee et al. [2]	Compare the efficacy of CGRP mAbs vs. nonspecific oral migraine preventives (NOEPs)	Systematic review and meta-analysis	Databases searched for class I or II RCTs comparing CGRP mAbs or NOEPs versus placebo for adult migraine prevention. Primary outcomes were monthly migraine days (MMD) or moderate to severe headache days.	CGRP mAbs are more effective than placebo with a small effect size; comparable efficacy to topiramate and divalproex but better tolerated. Supports use as first-line preventives.
Lu et al. [3]	Assess the myofascial release (MFR) effectiveness on headache pain intensity and disability	Systematic review and meta-analysis	Searched eight databases as of September 15, 2023. Evaluated risk of bias using the Cochrane RoB 2 tool.	MFR effectively reduces pain and disability in TTH and CGH. Inconsistent evidence for migraine.
Deng et al. [5]	Compare outcomes of lasmiditan, rimegepant, ubrogepant, and zavegepant for acute migraine management	Systematic review and network meta-analysis	Searched four electronic databases until August 31, 2023. The risk of bias was evaluated using the Cochrane tool, and the certainty of evidence was assessed via the CINeMA approach.	Rimegepant and ubrogepant show favorable efficacy and tolerability. Lasmiditan is effective but has a higher risk of adverse events.
Fernández-Bravo-Rodrigo et al. [6]	Assess clinical effectiveness and safety of erenumab for reducing migraine intensity and frequency in real-world settings.	Systematic review and meta-analysis	A systematic search of PubMed, Scopus, Web of Science, and the Cochrane Library from inception to December 2023. Included studies estimating real-world effects of erenumab.	Erenumab effectively reduces migraine intensity and frequency, showing strong real-world effectiveness and safety.
Suresh et al. [7]	Evaluate the safety and efficacy of zavegepant for acute migraine attacks	Systematic review and meta-analysis	Comprehensive search across various databases up to June 26, 2023, focusing on RCTs of Zavegepant's efficacy and safety in treating acute migraine. The primary outcome measured was freedom from pain at two hours postdose. Safety outcomes are assessed based on adverse event incidences.	Zavegepant is significantly more effective than placebo at relieving acute migraine and associated symptoms but with higher adverse events.
Kirkland et al. [8]	Assess comparative effectiveness and safety of parenteral agents for acute migraine pain reduction in emergency settings	Systematic review and network analysis	Searched nine electronic databases and gray literature sources. Included randomized clinical trials on parenteral agents for acute migraine pain. Protocol registered with PROSPERO (CRD42018100096).	Combination therapy and monotherapy with metoclopramide or neuroleptics are effective for managing acute migraine pain in emergency settings. Associated with adverse events like akathisia.

In evaluating the methodological quality of the selected studies included in this systematic review, the AMSTAR (A Measurement Tool to Assess Systematic Reviews) criteria were utilized to ensure a thorough assessment. The AMSTAR framework provides a comprehensive set of 11 criteria designed to evaluate the rigor and transparency of systematic reviews and meta-analyses. These criteria encompass aspects such as the establishment of an a priori design, the execution of duplicate study selection and data extraction, the comprehensiveness of literature searches, the inclusion of published and unpublished studies, and the assessment of publication bias. The following table summarizes how each study aligns with these criteria, highlighting areas of strength and potential gaps in methodological rigor. This structured evaluation aids in discerning the robustness of the evidence presented and in identifying the consistency of methodological practices across the included studies. A detailed summary of how each study meets these criteria is provided in Table 2.

**Table 2:** The AMSTAR framework provides a comprehensive set of 11 criteria designed to evaluate the rigor and transparency of systematic reviews and meta-analyses. AMSTAR: A Measurement Tool to Assess Systematic Reviews

AMSTAR Criteria	Robblee et al. [2]	Lu et al. [3]	Deng et al. [5]	Fernández-Bravo-Rodrigo et al. [6]	Suresh et al. [7]	Kirkland et al. [8]
A priori design established	Yes	Yes	Yes	Yes	No	Yes
Duplicate study selection and data extraction	Unclear	Yes	Yes	Partial	No	Yes
Comprehensive literature search performed	Yes	Yes	Yes	Yes	Yes	Yes
Status of publication as inclusion criterion	No	No	No	No	No	No
List of studies (included and excluded) provided	Partially	Yes	Yes	Yes	No	Partial
Characteristics of included studies provided	Yes	Yes	Yes	Yes	Partial	Yes
Assessment of scientific quality of included studies	Partially	Yes	Yes	Yes	No	Yes
Scientific quality used in formulating conclusions	Yes	Yes	Yes	Yes	No	Yes
Appropriate methods to combine study findings	Yes	Yes	Yes	Yes	Yes	Yes
Likelihood of publication bias assessed	No	Yes	Yes	No	No	No
Conflict of interest stated	Yes	No	No	No	No	No

Discussion

Our systematic review meticulously examined the efficacy and safety of various therapeutic interventions for migraine, drawing insights from recent high-quality studies. Notably, the meta-analysis by Lu et al. [3] demonstrated the effectiveness of myofascial release (MFR) in reducing pain and disability in patients with tension-type and cervicogenic headaches, although the results were less clear for migraine. Meanwhile, Deng et al.'s [5] network meta-analysis provided a comparative perspective, highlighting the favorable efficacy and tolerability of rimegepant and ubrogepant over lasmiditan, which, despite its effectiveness, presented a higher risk of adverse events. Fernández-Bravo-Rodrigo et al.’s [6] findings underscored the strong real-world effectiveness and safety of erenumab in reducing migraine frequency and intensity, reinforcing its clinical utility.

In acute settings, Suresh et al.’s [7] study noted that zavegepant significantly outperformed placebo in managing acute migraine attacks, albeit with an increased incidence of adverse events such as dysgeusia. Kirkland et al.’s research [8] into parenteral treatments in emergency scenarios pointed to the efficacy of combination therapies and monotherapy with agents like metoclopramide or neuroleptics, although these were also linked to notable side effects like akathisia. Lastly, Robblee et al.’s [2] analysis suggested that CGRP monoclonal antibodies (mAbs) were more effective than both placebo and traditional nonspecific oral migraine preventives, with a superior tolerance profile, supporting their use as first-line preventives. Collectively, these studies contribute robust data supporting various interventions while also highlighting the need for careful consideration of their safety profiles.

Our systematic review's findings align with and expand upon the existing literature on migraine management, reflecting both progress in therapeutic approaches and ongoing challenges in treatment efficacy and safety. For instance, the efficacy of CGRP antagonists like those studied by Robblee et al. resonates with emerging trends in migraine prophylaxis, where CGRP-targeting treatments are increasingly recognized for their potential to reduce migraine frequency with relatively favorable safety profiles, as evidenced in broader clinical practice and prior studies [9]. Similarly, the positive outcomes associated with myofascial release in Lu et al.’s analysis complement existing research advocating for integrated physical therapies in headache management, underscoring their value in a multi-modal treatment strategy [10].

Conversely, the concerns regarding the safety profiles of treatments like lasmiditan and zavegepant, noted in the studies by Xinxin Deng et al. and Suresh et al., echo the broader literature's emphasis on the need for a balanced approach to migraine treatment that considers both efficacy and potential adverse effects [11]. This aspect is particularly crucial as migraine treatments transition from acute relief to preventive care. Our findings support the ongoing shift toward personalized medicine in migraine management, advocating for treatment choices that are tailored to individual patient profiles, a theme that is increasingly prevalent in contemporary research [12]. This contextualization not only situates our review within the existing body of knowledge but also highlights its contribution to advancing the understanding of effective migraine management strategies [13].

The findings from our systematic review have significant implications for clinical practice in migraine management. The demonstrated efficacy of CGRP monoclonal antibodies and specific non-pharmacological treatments like myofascial release suggests a shift toward more personalized and targeted therapies [14]. For instance, the effectiveness of erenumab in reducing migraine frequency and intensity can guide clinicians toward prescribing it as a first-line preventive treatment, especially for patients who have not responded well to traditional therapies [15]. However, the safety concerns associated with treatments like lasmiditan and zavegepant necessitate a cautious approach, emphasizing the need to balance efficacy with potential side effects. These findings highlight the importance of considering individual patient profiles, including their past treatment responses and preference for certain treatment modalities, when devising treatment plans [16].

The strengths of this systematic review lie in its comprehensive and methodologically rigorous approach, including a broad search of multiple databases and the application of strict criteria for study inclusion and data extraction [17]. This robustness allows for a reliable synthesis of current evidence on migraine treatments. However, the review also faces limitations due to the heterogeneity of the studies included, particularly in terms of study design, participant demographics, and treatment protocols, which may affect the generalizability of the findings. In addition, most of the included studies focus primarily on adult populations, potentially limiting the applicability of the results to pediatric or elderly populations.

This review has identified several novel insights into migraine management, particularly the potential of CGRP monoclonal antibodies as a dominant preventive treatment and the effectiveness of myofascial release in managing chronic migraine symptoms [18]. These findings suggest that combining pharmacological and non-pharmacological treatments could enhance overall treatment efficacy and patient satisfaction. In addition, the review underscores the evolving trend toward more personalized migraine management strategies, where treatments are tailored based on specific patient characteristics and needs, setting a new direction for future clinical practice and research [19]. Moreover, the methodological innovations in this review, such as the application of network meta-analysis in comparing multiple treatment options across different studies, provide a more nuanced understanding of how various treatments compare in real-world settings.

Our systematic review has highlighted several gaps in current research on migraine management, pointing to critical areas for future studies. One notable gap is the long-term efficacy and safety of new pharmacological treatments, such as CGRP monoclonal antibodies [20]. While promising, their long-term impact remains under-explored, necessitating longitudinal studies to assess sustained effects and potential long-term side effects [21]. Another area for future research involves examining the effectiveness of treatments across diverse patient subgroups, including variations based on demographic factors like age, sex, and race, which could influence treatment outcomes. In addition, there is a need to investigate the impact of combining therapies, such as integrating pharmacological treatments with non-pharmacological interventions like myofascial release or cognitive behavioral therapy, to determine synergistic effects that might enhance overall treatment efficacy. Addressing these gaps will not only enhance our understanding of migraine management but also refine treatment protocols to optimize patient outcomes [22].

The findings from this systematic review have significant practical and clinical implications that could influence future clinical guidelines, patient care strategies, and health policy. The efficacy of newer treatments like CGRP monoclonal antibodies suggests that updating clinical guidelines to include these as recommended therapies for preventive migraine management might be warranted [20]. Such changes could lead to better patient outcomes and more personalized treatment plans. In terms of patient care strategies, our findings emphasize the importance of individualized treatment approaches, taking into account patient preferences and specific migraine characteristics. Health policies may need to adapt to ensure broader accessibility and affordability of these newer treatments, which could involve negotiating drug prices or revising insurance coverage policies to include comprehensive migraine management options.

Moreover, the practical implementation of these treatments in clinical practice must consider factors such as cost, accessibility, and patient education. For instance, while CGRP monoclonal antibodies offer a promising treatment avenue, their high cost might limit accessibility for many patients without appropriate insurance coverage [23]. Healthcare systems should consider strategies to mitigate these barriers, potentially through subsidized programs or educational initiatives aimed at informing both healthcare providers and patients about the most effective and sustainable migraine management practices. This comprehensive approach will not only enhance treatment efficacy but also improve overall patient well-being and quality of life [24,25].

## Conclusions

This systematic review has critically assessed the current landscape of therapeutic interventions for migraine, revealing significant advances in both pharmacological and non-pharmacological treatments. Our findings confirm the efficacy of CGRP monoclonal antibodies and myofascial release, highlighting their potential as first-line and adjunctive treatments, respectively. The review underscores the importance of personalized treatment strategies, balancing efficacy with safety, particularly given the diverse patient responses and potential adverse effects associated with newer pharmacological agents. Moving forward, it is imperative that clinical guidelines be updated to reflect these insights, ensuring that migraine management is both effective and patient-centered. Future research should continue to explore the long-term effects of these therapies and the potential benefits of their integration, aiming to optimize outcomes for all migraine sufferers.
